# WTF?! Covid-19, indignation, and the internet

**DOI:** 10.1007/s11097-023-09889-z

**Published:** 2023-02-01

**Authors:** Lucy Osler

**Affiliations:** Cardiff University, Cardiff, Wales

**Keywords:** Indignation, Disbelief, Surprise, Moral anger, Covid-19, Social media

## Abstract

The Covid-19 pandemic has fuelled indignation. People have been indignant about the breaking of lockdown rules, about the mistakes and deficiencies of government pandemic policies, about enforced mask-wearing, about vaccination programmes (or lack thereof), about lack of care with regards vulnerable individuals, and more. Indeed, indignation seems to have been particularly prevalent on social media platforms such as Twitter and Facebook, where indignant remarks are often accompanied by variations on the hashtag #WTF?! In this paper, I explore indignation’s distinctive character as a form of moral anger, in particular suggesting that what is characteristic of indignation is not only that it discloses moral injustices but betrays our disbelief at the very occurrence of the offence. Having outlined the character of indignation, I consider how the structure of indignation impacts how we *do*, *respond* to, and *receive* indignation. I explore indignation in action, so to speak, in the context of Covid-19, with a particular emphasis on how indignation occurs ‘on the internet’.

## Introduction

They haven’t imposed a lockdown yet?? They’ve imposed another lockdown?? The prime minister was attending lockdown parties while I attended my friend’s funeral on Zoom – WTF?! How dare you put vulnerable people at risk?? You can’t force me to get vaccinated! I cannot believe I *still* have to wear a mask! I can’t even visit my grandmother in her nursing home?!

The covid-19 pandemic has fuelled indignation (Li et al., 2020; Vindegaard & Benros, 2020). People have been indignant about the breaking of lockdown rules, about the mistakes and deficiencies of government pandemic policies, about enforced mask-wearing, about vaccination programmes (or lack thereof), about lack of care with regards vulnerable individuals, and more. Indeed, indignation seems to have been particularly prevalent on social media platforms such as Twitter and Facebook, where indignant remarks are often accompanied by variations on the hashtag #WTF?! (Yu et al., 2021; Farrell et al., 2020). It seems appropriate, therefore, to list indignation alongside other so-called ‘emotions of the pandemic’, such as grief (Richardson et al., 2021), loneliness (Osler, 2022a), shame (Dolezal et al., 2021), envy (Protasi, 2021), and anxiety (Trigg, 2022).

Indignation, though, is a relatively under-theorised emotion in philosophy. On the rare occasions when it is mentioned, indignation is typically raised in the context of anger. There is, however, no consensus about what indignation’s relationship to anger is. Some take indignation to be a distinct emotion from anger (e.g., Kriegal, 2022), while others suggest it is a *type* of anger (often without then specifying what distinguishes it from other types of anger) (e.g., Cherry, 2014; Silva, 2021a), and others take the two words to be synonymous (e.g., Nussbaum, 2016). Prompted by the recent proliferation of indignation during the Covid-19 pandemic, I want to place indignation front and centre and explore what might be distinctive about this emotion. As part of this philosophical exploration, I suggest that previous discussions of indignation have overlooked or downplayed a key feature of this emotion, namely *disbelief*. I take it that we feel indignation when something occurs that we take to be morally wrong which takes us aback. Indignation, as I understand it, is an emotion of affront and incredulity. As such, I draw attention to how indignation not only discloses something as morally wrong but also betrays our own surprise, even disbelief, at the *very occurrence of the offence*.

I am not only interested in what indignation *is* but what indignation *does*. With a more robust understanding of what is distinctive about indignation, I consider how the structure of indignation impacts how we *do*, *respond* to, and *receive* indignation. In particular, I explore: (i) how indignation can be an effective tool for self-disclosure and, as such, can work to build and sustain social connection, (ii) why indignation is a fertile ground for virtue signalling, (iii) how expressions of indignation can often strike us as naïve and even downright offensive, and (iv) the potential political and moral power of holding onto indignation. I explore indignation in action, so to speak, in the context of Covid-19, with a particular emphasis on how indignation occurs ‘on the internet’.

In Section 2, in order to situate the discussion, I provide a brief background to the philosophical discussion of anger. In Section 3, I set out what I take to be the key features of indignation: that it primarily targets an offence (not an offender), the offence is disclosed as a moral offence, it is a ‘self-regarding’ emotion, one’s own harm is accidental, and the occurrence of the injustice is experienced as surprising, even begging belief. Having sketched what is distinctive about indignation, in Section 4, I explore how indignation has been used, received, and responded to during the Covid-19 pandemic, particularly expressions of indignation on the internet. In doing so, I shed light on the broader character of indignation, especially highlighting why indignation can often be experienced as somewhat distasteful, naïve, and even offensive due to its relationship with disbelief. I conclude, however, with a partial defence of indignation in certain settings.

## A brief introduction to anger

Given that indignation is almost exclusively mentioned in the context of anger, it seems fitting to start with a very brief discussion of anger. With this broad background of anger in place, I turn to John Drummond’s account of indignation as our starting point for thinking about what might be distinct about indignation that distinguishes it from other anger-like experiences such as rage, resentment, and other forms of so-called moral anger.

### Anger

Unlike indignation, a lot of philosophical work has been devoted to anger. There is general agreement (unusual in philosophy) that anger is an emotional response to (or a disclosure of) a perceived offence or injustice. Say I am angry at you for taking the last chip. In being angry, my anger discloses what you have done as unfair or wrong. Here, unfortunately, the agreement peters out. There is much debate about the nature of anger, especially questions about whether it must necessarily involve bodily feeling, whether it is essentially moral, and whether it is retributive in nature. In short, my position on these issues is: yes, no, and no. I will briefly set out my own views on these points. I take it that one could disagree with me on one or more of these issues and still find something of interest in my account of indignation. However, I think it is helpful to understand some of the main points of contention in relation to anger broadly construed before we turn out attention specifically to indignation.

While I will not defend my philosophy of emotion here, I (broadly) follow Helm (2001) in taking emotions to be “felt evaluations”.^1^ Emotions are feelings that disclose the world in a certain way to us. The cliff is revealed to me *as* dangerous through my fearful apprehension of getting close to the edge, the chip-taker is revealed to me *as* doing something offensive through the welling up of anger I experience when they grab the last chip. The feelings are both intentional (they are about something in the world) and evaluative (they reveal the world as being a particular way based on my own concerns). What makes an emotion a particular type is that it shares a common evaluative characteristic, or formal object, with others (Kenny, 1963; Teroni, 2007). For instance, the formal object of fear is danger, the formal object of anger is offence. Each emotion also has a *particular* object, that to which it is directed in the instance, described as the *target*. In watching you take the last chip, the target of my anger is you. In adopting this general view of emotions, I align myself with many phenomenological accounts of emotion and view emotions as an essentially embodied and affective affair.

Some think that anger is an intrinsically moral emotion (e.g., Nussbaum, 2016), while others allow for anger that involves a perceived offence that is not a moral offence (e.g., Drummond, 2017; Cherry, 2014). I want to leave the door open for non-moral anger: anger that involves a felt evaluation of something being offensive or wrong but not necessarily morally offensive or wrong. This allows us to account for common-or-garden varieties of anger such as the anger I experience when I stub my toe on the table, when I am angry at myself for forgetting to bring my phone charger to work, feeling angry at you for being in the bathroom when I am desperate for the loo, being angry that there is a thunderstorm when I want to go wild swimming.

As such, I, like others, am inclined to take anger to be an umbrella term for a family of related emotions (e.g., Bell, 2009; Cherry, 2022; Pettigrove, 2012; Silva, 2021a; Srinivasan, 2018). As Flanagan (2018, x) notes: “In English, one might think that these are some of the species of anger: rage, outrage, hatred, fury, indignation, irritation, frustration, resentment, prissiness, impatience, envy, jealously, revenge, and vengeance”. We might even add to this list: annoyance, contempt, disdain, exasperation, and likely more. Different types of anger all share the formal object ‘offence’ but disclose this formal object in slightly different ways. Now it is likely that many of these different types of anger overlap in various ways and some may well be better thought of as linguistic synonyms rather than distinct forms of anger. Nevertheless, by exploring the specific way that an anger-type emotion discloses an offence, we can capture what is distinctive about these types of anger. Different types of anger might also have different functions, roles, and reception as well. As such, refining the profile of different forms of anger not only is of philosophical interest but also of practical interest and import.

Traditionally, anger has been taken to not only involve the evaluation of an offence or injustice, but to have an accompanying desire for revenge or payback for that perceived offence. Anger, then, has been taken to be essentially *retributive*. In the words of Aristotle, anger is “a desire accompanied by pain, for a conspicuous revenge for a conspicuous slight at the hands of men who have no call to slight oneself or one’s friend” (Aristotle, 1984, Rhet. 1378, 31–34). While anger’s retributive nature has long been assumed, recently there has been a call to recognise that anger does not necessarily involve a desire for retribution. To borrow Laura Silva’s (2021a) example, if I am angry at you as my friend for not having supported me while I was going through a difficult separation, I do not harbour any desire for you to suffer or to take revenge on you. Rather, what I want is for you to *recognise* the harm that you have caused me and see my anger as justified. We find this emphasis on anger involving recognition also in the work of Myisha Cherry (2022) and Amia Srinivasan (2018). I think this move away from viewing anger as essentially retributive is the right one and likely a helpful way to further distinguish different kinds of anger from one another. However, in acknowledging different forms of anger, caution should be adopted in the move to unseat one necessary condition for all types of anger if this leads us to adopting another universal condition. As I will argue below, while I endorse the move towards highlighting the role recognition might play in many cases and types of anger, I will suggest that indignation is often more concerned with recognition by a broader community than a specific offender.

### Moral anger

Recently, it has been argued that we should embrace a new subclass of anger: moral anger (e.g., Cherry, 2014, 2021; Srinivasan, 2018). Moral anger reveals a specifically moral offence. To use an example from Cherry (2014), one might be morally angry if you believe you have been fired based on racial discrimination, but one can only be angry (not morally angry) if you believe you have been fired due to your own failures. These contemporary discussions of moral anger have, in particular, emphasised the appropriateness of anger (Srinivasan, 2018), as well as its motivational and productive features (Cherry, 2014, 2021; Silva, 2021a, b; Tanesini, 2021).^2^ In doing so, such debates have persuasively argued for the recognition of the virtues of moral anger in the face of moral injustices such as racism. As indicated above, in order to preserve cases of anger that do not target specifically moral injustices or offences, I welcome and endorse this specific category of moral anger.

Interestingly for our purposes, Cherry (2014) and Silva (2021a) both name indignation as an example of moral anger, alongside resentment. However, neither author suggests what might be distinctive of indignation as a form of moral anger that would distinguish it from other kinds of (moral) anger. As is clear from the analysis below, I agree that indignation is a specifically moral kind of anger. However, by recognising the category ‘moral anger’, we are put under pressure to say something more to justify the claim that indignation is not only a type of anger but more specifically a distinctive kind of moral anger.

## The character of indignation

I now outline what I take to be the characteristic features of indignation. Many of these features draw on John Drummond’s account of indignation from his 2017 paper “Anger and Indignation”. However, I suggest an additional component that Drummond himself does not take to be central to indignation: surprised disbelief. In his account, Drummond persuasively sets out indignation as a distinct kind of emotion but its distinction from ‘anger’ primarily hangs on indignation’s status as a *moral* emotion. Importantly, by highlighting disbelief as a core feature of indignation, I think we are able to demarcate indignation not only from ‘anger’ broadly speaking but as a specific type of moral anger.

Note that in this discussion, I argue that indignation has certain characteristics that warrant it being regarded as a specific kind of anger-emotion. Nevertheless, it may well be that other types of anger have overlapping characteristics with indignation and even that there are synonyms for indignation in various languages. For example, I take it that some examples of what is dubbed ‘moral outrage’ might overlap with the kind of emotion I am referring to by the label ‘indignation’. Moral outrage, as I argue is the case for indignation, can also be associated with the experience of being morally affronted or taken aback by the occurrence of a moral injustice. The aim of this analysis, however, is not to consider what words are best used to pick out the experience of indignation in ordinary language but to consider the structure of this emotion. I take the examples set out at the beginning of this paper to be illustrative of indignation – one feels indignation in the face of something morally offensive that takes us aback, where the very occurrence of the offence is experienced in a state of incredibility. I, therefore, take indignation to be epitomised by experiences where we might proclaim “How dare you…?” or “I cannot believe that…!” or, in internet parlance, “WTF?!”.

### Targets a moral, social, or political offence

As mentioned above, we can experience anger in relation to non-moral offences. I might be angry that it has rained without positing the fact that it is raining as a moral, political, or social injustice. Rather, I experience the rain as a frustration of my own goals. Indignation, on the other hand, discloses something as morally offensive to me.^3^ For instance, I might be indignant that companies producing PPP equipment for hospitals during Covid-19 were selected on the basis of cronyism, I might be indignant that people have not been careful about maintaining a two-meter distance in supermarkets. In being indignant, I take the offence as being against what I think the moral, social, or political situation *should* be.

### Targets the offence not the offender

In many cases of anger (or anger-type emotions), we are angry *at* someone or some group. I am angry *at* Lana for taking my favourite jumper, I am angry *at* my family for bickering. My anger appears to pick out both the offence (e.g., stealing) but also the offender (e.g., Lana). Anger, in this sense, is often a personal affair. I am not just angry at the offence of stealing but *at* Lana *for* stealing. Landweer (2020, 449) nicely captures this as follows: “The phrase “to be angry with someone (for something)” suggests that anger has one or more persons as a condensation area”. This fits with P.F. Strawson’s notion of anger as a reactive attitude. According to Strawson (1962), emotions such as anger are *reactions* to other moral agents that signal that they have flouted a norm but also, importantly, treats that agent as capable of reform. When we are angry at someone our reaction signals that their behaviour has breached a norm but also presents them with an opportunity to re-enter the community. Anger, as a reactive attitude, holds others to account.

Indignation, though, does not necessarily target a specific offender. If Axel is indignant about the lockdown rules in the UK, his indignation (at least primarily) discloses the perceived injustice of the curtailment of freedoms rather than a particular offender (Drummond, 2017; Landweer, 2020). In indignation, “[w]e name not the wrongdoer or cause, but the harm, the wrong or offence, or the situation giving rise to the wrong or the offence” (Drummond, 2017, 20). Interestingly, in disclosing an offence rather than an offender, it is not clear that indignation desires revenge upon an *offender*, as it is often not clear who that offender might be. In a similar vein, it is also not clear that indignation obviously calls for, or could be satisfied by, an apology. In expressing indignation about lockdown rules, Axel is not demanding that anyone apologises for the injustice that he identifies. It may be the case that indignation more broadly aims for the ‘rectification’ of the offence (though I will not explore this view here).

What about cases where there is a specific individual involved? For instance, if I am indignant at my neighbour’s flaunting of lockdown rules? Even here, where the offence arises from the action of a specific person, I, like Drummond, think indignation primarily picks out the perceived offence (e.g., putting others at risk, violating the ‘social order’). The individual is not so much perceived as *the offender* but as the personification of the offence itself. In this regard, we might say that indignation is a “cooler” emotion than other types of anger because it aims at injustice per se rather than at a personal offender (Drummond, 2017; Landweer, 2020; Kriegel, 2022). Below, I suggest that recognising disbelief as a component of indignation also helps clarify why indignation seems to primarily pick out the offence rather than the offender. In short, I argue that part of the experience of being indignant is being surprised that the offence could have happened, thus being taken aback by the offence occurring at all, not simply that a particular offender or offenders did something offensive.

### Self-regarding

When we are indignant, we adopt a position of superiority. As Drummond puts it, we are “self-aware in a particular way”, the indignant person is “pre-reflectively – and in some cases reflectively – aware of herself as in a morally superior position” (2017, 22). When we feel indignation, we feel that something ‘*should not be so*’– not simply because we don’t like the state of affairs but we take ourselves to have correctly identified the occurrence of some kind of moral injustice or offence. Jayce might be indignant about certain countries offering people booster vaccines before much of the world’s population has yet to receive a first shot, taking herself to be right that this is a case of gross inequality. Ruby might be indignant about being told to wear a mask, seeing herself as correctly defending her right to self-determination. Tika might be indignant that people are breaking lockdown, positioning themselves as being in the morally superior position of not being someone who would break the law.

This does not mean that the person is, in fact, in a position of moral superiority. A person might be indignant about a situation without being fully aware of all the facts – e.g., Fran might be indignant about a someone not wearing a mask, taking themselves to be rightly upholding protective social measures, while not aware that the person in question cannot wear a mask for medical reasons. Or someone might be hypocritically indignant – e.g., Teri might be indignant about lockdown parties, despite having held a large gathering herself the other week that they conveniently seem to have forgotten about.^4^ Nor does this positioning have to take the form of an explicit judgment about what moral norm has been violated but rather a person “might just recognize – perceive as it were – the injustice of the action or situation about which she is indignant” and consider herself as being in a position from which it is justifiable to be indignant (Drummond, 2017, 22). Descartes (1984, 398) goes so far to suggest that when we feel indignation, we can feel “admiration” for ourselves that “we would not do the like”.

Emotions that take oneself as the target of the emotion are described as “self-conscious” emotions. For instance, if I feel self-directed shame, the target of my shame is me, I am ashamed *of* myself (Salice & Sánchez, 2016), or in cases of self-directed pride, I am proud *of* myself (Sánchez & Salice, 2022). Indignation does not take the subject of indignation as the target of the emotion; one is not indignant at or about oneself. While indignation looks self-involving in some way, it is not, then, a self-conscious emotion. Rather, we might describe indignation as a self-*regarding* emotion. Our indignation does not simply disclose something as offensive; it also involves a self-judgment about having the moral standing to proclaim that thing morally offensive. Contrast this to cases of non-moral anger where I might be embarrassed or amused by my own anger.

### Own harm is not necessary

As both Drummond (2017) and Kriegel (2022) note, another reason why we might think of indignation as a ‘cooler’ emotion than other forms of anger, such as rage, is that when we are indignant, our own involvement is “accidental”. We can, of course, be indignant in relation to things that directly concern oneself. I might be indignant that I have to teach face-to-face during a pandemic, while others are allowed to teach online. However, I might also be indignant that others are forced to teach face-to-face at risk to their own health, even when I am allowed to teach virtually. Indignation is not, then, necessarily vicarious but it can be. Even when I experience indignation in relation to an offence that personally affects me, there is a certain “detachment of one’s felt evaluation from the specificity of one’s own involvement in the situation” (Kriegel 2022, 10) – I judge the offence to be wrong whether it happens to impact me or not. We might talk of indignation as being an ‘allyship’ anger, a form of anger that can be had ‘on behalf of’ others.

### Disbelief

Although Hilge Landweer (2020) takes the corporeality of anger and indignation to be identical, Drummond (2017) highlights certain physiological differences between anger and indignation. Anger often involves a contraction of the body (e.g., frowns, clenched fists, tightening lips and jaw):

In indignation, the face manifests a set of changes similar to what we find in surprise or shock. The eyes widen with the eyebrows and eyelids pulled up, and the mouth opens in a more circular than squarish shape. But the surprise is negatively valenced; we experience a feeling of shock at an affront or brazen wrong of some kind, and we cannot quite believe that what we have witnessed (directly, through media, or through testimony) has in fact happened. (Drummond, 2017, 19)

While I think Drummond is right to identify this feature of indignation, he makes little of it. He merely notes that the embodied differences between anger and indignation suggest, but do not establish, more significant differences between these emotions.

The embodied difference that Drummond points out, however, goes beyond a physiological difference to an important phenomenological one: we experience indignation when we not only perceive something to be morally wrong but *the occurrence of this injustice runs counter to our expectations*. Part of what it is to feel indignation, as opposed to other anger-type experiences, is the experience of being taken back. As Drummond puts it, we experience indignation when “we cannot quite believe what we have witnessed” – we experience indignation when we are surprised by, even incredulous of, the very occurrence of the offence. It sounds odd to say that I feel indignant that one of my partners is late, when I fully expected them to be so because they are always late. I might be frustrated, annoyed, or enraged in this situation, but it seems odd to describe this as leading to indignation.

I suggest, then, that indignation is an emotion that involves negatively valanced surprise or disbelief.^5^^,^^6^ Daniel Dennett (2001, 982) describes surprise as “a telling betrayal of the subject’s *having expected something else*”. Indignation not only reveals our moral values and own self-regard as being in a position of moral superiority, but it also reveals our expectations about the world and how our sense of reality is disrupted by the occurrence of that offence. We react with indignation not only when we witness an offence but the occurrence of the offence is in tension with how we think the world is. Note that the disbelief felt is not a disbelief that the occurrence has actually happened (it is not doubting the occurrence of the offence) but a disbelief that it could have happened.

In indignation, we not only take it that an offence *should not* have happened, but we are surprised that it *could have* happened at all. If Ulla is indignant that people are not wearing masks, that others are violating lockdown rules is in tension with her understanding of the world as somewhere where people would rather put on a piece of uncomfortable cloth than risk spreading a potentially deadly virus. Ulla’s indignation involves a disbelief that people would wilfully violate lockdown rules. Her indignation does not only ask ‘how could they do this?’ but ‘how could it be this way?’. The very breaking of lockdown rules grates against her idea about what people would do in a state of pandemic emergency. Part of what is distinctive about indignation is precisely that we experience it when we witness offences that unsettle our taken for granted attitude about what kind of world we live in. This, I think, helps explain why indignation is primarily directed at the offence not the offender(s), because what begs our belief is that the offence happened at all, that its occurrence does not sit comfortably with our broader idea of how we take the world to be. Indignation is felt not only when we apprehend a moral offence but when we assumed that things would be otherwise.

Now, this is not to suggest that we can only experience indignation when we perceive a moral offence whose occurrence runs contrary to our *explicit* expectations. Gene might be indignant about vaccine mandates while never having explicitly held beliefs about the morality of vaccination programmes. Rather, their implicit expectation of having their bodily autonomy respected by the government is upset by the mandate. Expectations, then, can be explicit or tacit, and are often informed by conventions, norms, and habit (Judge, 2018; Stockdale, 2022).

Can we not, though, feel indignation in circumstances where our expectations are confirmed, rather than contradicted? What about when I feel indignant that the government has been hosting parties during lockdown against its own lockdown regulations, when I already expect the government to treat its own members as somehow above the laws that it sets down for the rest of its citizens? In such a case, we might suppose that I am not surprised by instances of governmental hypocrisy and corruption. Indeed, we might go so far to suggest that I actually expect the government to act in such a way. Does this not undermine the idea that surprised disbelief is a feature of indignation?

One response might be that while we might believe that a government is corrupt, or at the least is prone to hypocrisy, while still being surprised about the occurrence of a specific event. An individual could be distrustful of their government, even outrightly believe that the government holds itself as above its own laws, while being taken aback when it comes to light that many of the government’s own members were in frequent and clear breach of lockdown rules. We can make a distinction, then, between our expectations in general and our expectations about a specific situation. For instance, we are often shocked by specific instances of sexist and racist behaviour even when we know all too well that such behaviour proliferates in general – the surprise can be about it coming from *this* quarter or in *this* way.^7^

However, I think a more common case might be that even in the face of certain moral offences, we might fail to assimilate the occurrence of such offences into our understanding of what the world is like. We might remain incredulous that we live in a world where the persistence of government corruption would be tolerated or allowed to continue. The surprise in question here is a negatively valanced, affectively charged disbelief in the face of this persistence. In our indignation, despite evidence that speaks to the contrary, we remain appalled and taken aback that such a thing *could* (continue to) happen.^8^

We might suppose, however, that if these offences happen frequently enough, that my indignation about government gatherings during lockdown might transition into something more like resentment or rage – where I resent or am enraged by the government for systematically violating lockdown regulations, but I no longer experience these revelations as surprising or begging belief. Rather, over time, my understanding of the world assimilates to one in which I fully expect and believe in the corruption and hypocrisy of a government. It is difficult to harbour indignation, for the very process of harbouring our affront can prompt a reassessment of how we take the world to be, leading to the evaporation of our disbelief. This does not mean our anger about the offence must also evaporate. Indignation might sediment or morph into an on-going anger about an offence – where our disbelief at the occurrence of an offence disappears in favour of a deep-seated anger, rage, or resentment about this state of affairs. This, I think, helps capture that indignation, in being an emotion of negatively valanced surprise, is typically a short-lived emotion, whereas other forms of anger, such as resentment, might be emotions that we can hold onto for a long time. I will return, however, to the idea that we might hold onto our incredulity and our indignation in Section 4.4.

Where our worlds are in a state of upheaval and disarray, atypical policies, rules, behaviours, and beliefs abound that likely come into tension with how we took the world to be. Note that a pandemic, where many of our experiences and events fall outside of our usual habits and expectations, might be supposed fertile ground for encountering unexpected events and behaviour and, as such, prompting and feeding feelings of surprise, disbelief, and indignation.

## Indignation in action

Having attempted to clarify the characteristics of indignation, I now want to turn to what indignation *does* – how we use, receive, and respond to indignation. I want to explore how indignation unfolds in the wild, so to speak, due to its specific profile and why we might have seen a proliferation of indignation during the Covid-19 pandemic. I also want to analyse why expressions of indignation are often experienced as somewhat distasteful, as having the whiff of self-aggrandizement, naiveté and even privilege.

### Indignation and self-disclosure

When we express indignation, this discloses something about our moral values. In Section 2, I discussed the increased attention being given to the role that recognition plays in anger in terms of striving for recognition from an offender that what they have done has caused harm or offence. Considering indignation highlights another way in which forms of moral anger involve recognition, i.e., in terms of disclosing one’s moral commitments to an audience.^9^

In being morally self-disclosive, expressions of indignation can play a useful role in certain interpersonal interactions. We often drift towards friends and communities with whom we have a certain amount of normative overlap. An emotion that reveals one’s (continued) commitment to a set of moral norms, then, can help people generate and sustain social bonds with one another. Given the stress I’ve placed on disbelief as a component of indignation, I also think that expressing indignation not only serves as a statement about one’s morals but also discloses, perhaps more subtly, how one takes the world to be.^10^ One’s indignation over not being able to travel during a lockdown, not only signals that one finds this curtailment of freedom morally offensive but also that one assumes that one’s freedom to travel is secure and a violation of this is incredible. Indignation might, then, not only be morally self-disclosive but also world disclosive.

This self-disclosive feature might serve a particularly helpful function in online social spheres and even help account for why social media platforms often seem to be a hotspot for expressions of indignation.^11^ During various lockdown measures during the pandemic, many of our social interactions have moved onto internet-enabled platforms. However, when we go online, we might find ourselves on online platforms uncertain about how to find people like us, as well as uncertain about how to present oneself in the online sphere. Indignation is an efficient tool for situating a user as a holder of various moral (and political and social) values and perhaps even as experiencing the world being a particular way. Indignation, then, can help up situate ourselves as a social, moral, political being, signal our belonging to a certain community, as well as demonstrate our continued commitment to the values of our communities.

In a recent paper, Tanesini (2022) considers how certain design features of social media platforms promote the contagion of group-based anger, for instance, through the ability to like, share, and comment upon people’s online expressions of indignation. This suggests that not only might the signalling function of indignation be particularly effective on social media platforms but that these platforms might actively promote the proliferation of collective indignation. Social media platforms, then, might not only encourage emotions such as indignation that have this morally self-disclosive feature but also enflame the spread of such emotions.^12^ Ironically, then, an emotion that can function to support and sustain social connection in online spaces at a time when we are physically separated from one another, might also lead to increased levels of affective and collective polarisation.

Interestingly, this self-disclosive feature may, in times of crisis such as the Covid-19 pandemic, serve another purpose.^13^ Another feature of indignation is that it positions the indignant individual in a position of moral superiority, as in a place from which to judge. In moments of upheaval, when there is chronic uncertainty, it might also be reassuring to experience indignation, an expression of moral certainty that not only signals to others but also to oneself. To put it another way, in times of uncertainty, we might appreciate being an audience to our own indignation as it can create a reassuring sense of moral certainty when we are struggling with lack of control and surety.

### Indignation and virtue signalling

In highlighting how indignation might relate to its audience, we touch upon a concern that has previously been expressed about indignation (e.g., Drummond, 2017; Descartes, 1984; Nietzsche, 1989). Namely, that people who express indignation might be more concerned with what their indignation signals, rather than expressing genuine indignation about moral injustice. In the words of Descartes (1984, 398):

[I]ndignation is observed much more in those who wish to appear virtuous than in those who really are virtuous.

To situate this within a hot topic in philosophy and public discourse: we might be concerned that indignation lends itself to moral grandstanding (Tosi & Warmke, 2016) or virtue signalling (Levy, 2021).^14^

Tosi and Warmke (2016, 199) define moral grandstanding as an expression that makes “a contribution to moral discourse that aims to convince others that one is “morally respectable””. Moral grandstanding can be seen as another term for the more common phrase ‘virtue signalling’. The main motivation behind moral grandstanding is to convey one’s own virtue to an audience. According to Tosi and Warmke (2016), we should condemn moral grandstanding as a narcissistic and self-promoting exercise. Indeed, they go so far to describe moral grandstanding as “repugnant” and a case where “moral talk itself can become a form of bad behaviour” (2016, 198), as grandstanding turns one’s contribution to moral discourse into a “vanity project” (2016, 199). Other than a concern that moral grandstanding might be narcissistic, Tosi and Warmke (2016) suggest that it can have a malignant impact on public moral discourse. They outline a number of pernicious effects of moral grandstanding, including promoting excessive outrage and polarization, as well as a devaluation of moral talk.

The self-disclosive character of indignation appears to make it a ripe ground for virtue signalling, for in its very expression is the disclosure of one’s (alleged) moral commitments. Expressing indignation, then, might easily slide into moral grandstanding when the indignant person is primarily concerned with signalling their own moral commitments and, in doing so, appearing virtuous in the eyes of others. Moreover, indignation, in situating the indignant as in a position of moral superiority, might add to the impression that it is a self-aggrandizing emotion. Indignation, then, looks like it is not only *self*-regarding but disposed to also be *other*-regarding.

In the context of the Covid-19 pandemic, where much of our public moral discourse has taken place on social media platforms, we might have even more concern about indignation being used for virtue signalling. When expressions of indignation are posted to social platforms, we might be worried that this is all they are, *expressions*. One can imagine a person sitting comfortably at home enjoying their toast and marmite, while spouting off indignant tweets about the hypocrisy of governments, the shocking behaviour of people not social distancing, and so on. As Neil Levy nicely frames the potential worry:

Social media makes virtue signals easier to fake because it is very much harder to observe the involuntary concomitants of genuine emotion, and because it is harder to monitor behavior across time and in different contexts online. (Levy, 2021, 22)

Indeed, if indignation can be helpful for situating oneself in a particular community, we might suppose that the potential social benefit of indignation will further promote these instrumental expressions of indignation in online spheres – it not only signals one’s own virtue but can lead to feeling a sense of belonging with others ‘like you’.

A proliferation of indignation during the pandemic may well lead to suspicion and scepticism about how genuine these expressions of indignation really are. Moreover, following Tosi and Warmke (2016), if much indignation expressed online is virtue signalling, this could potentially have a long shadow. Our suspicion of indignation as a virulent form of virtue signalling might work to mask real expressions of indignation and rob indignation of its motivational and productive function as a way to highlight, and even demand rectification of, moral injustices. Expressions of online indignation also look like good candidates for what Nguyen and Williams (2020) dub “moral outrage porn”; expressions of moral anger that give a reader gratification without promoting authentic or productive engagement with moral discourse.

However, as is so often the case, things are not as simple as they appear on first pass. Levy (2021) has recently highlighted the potential virtues of virtue signalling. Levy points out the important epistemic role that agreement plays – we (typically) feel more confidence in our commitments when others agree with them – and argues that part and parcel of the function of moral discourse is signalling one’s commitment to norms. As such, he states that the claim that virtue signalling undermines moral discourse is on “very shaky ground” (Levy, 2021, 9555). Moreover, he suggests that emotions like moral anger are good signals, in part, because they are costly ones that are hard to fake. Even in the realm of social media, where we cannot know if the expression of indignation is accompanied by an authentic feeling of indignation, Levy astutely notes that if virtue signalling is about reputation, and that many users of Twitter and Facebook post under their real names, “there seems little reason to believe that a very significant proportion of virtue signallers are deceptive, even on social media” (Levy, 2021, 9559). While social media platforms might promote the expression of indignation and those posting might indeed be concerned with how their expressions signal their moral commitments, even their virtuousness, this does not necessarily render such expressions inauthentic nor a threat to moral discourse.

### Indignation and the political parvenu

Indignation can come across as a smug emotion, for indignation positions oneself as being morally superior. Perhaps worse, though, there are many occasions when someone’s indignation can strike us as insensitive, naïve, even downright offensive. Say that you are scrolling through your Twitter feed after a government announcement about the extension of a lockdown. On your feed is a tweet from Giles proclaiming their indignation about the government unlawfully and unjustifiably trapping them in their house – “The government thinks it has the right to lock us in our houses – WTF??”. His indignation leaves you with a bitter taste in your mouth. But why?

First, you might simply disagree with Giles’ indignation, refuting that being put into a lockdown during a pandemic is something morally wrong. Let us suppose, though, that you agree with Giles that there is something morally wrong with the lockdown. From this stance, you still might experience his indignation as distasteful for a second, more nuanced, reason. Indignation is not a subtle emotion. In being indignant, Giles discloses his perception of being forced into lockdown as morally wrong. Moreover, it discloses the moral certainty that Giles has about this. One might be suspicious of such robust moral certainty in the face of complex moral, social, and political circumstances. Even if sympathetic to the idea that being put into a lockdown is (or can be) morally wrong, you might think that Giles’ indignation represents the situation in too simplistic a manner.

Let us suppose, though, that you agree with Giles that the lockdown is a moral offence and that it is clearly so. Even where you agree with the moral assessment of Giles’ indignation, I think his indignation might still strike us as inappropriate. I think this is due to the fact that indignation not only discloses the indignant person’s perception of a moral injustice but also their disbelief at its occurrence. Giles’ indignation about being forced into lockdown by the government not only discloses that he thinks it a moral injustice that the government can exercise this power over him but also his incredulity that the government can and will do so. His indignation seems to not just ‘*why* would they do this?!’ but ‘*how* could they do this?!’ and his expectation that the government will not interfere with his freedom is, in part, born out of being a citizen whose freedoms are not typically infringed upon in this way.

Someone who has experienced a history of oppression at the hands of the same government may think the lockdown violates their freedom as a citizen but may not be surprised at what they might perceive as yet another predictable incursion of the government upon their autonomy and self-determination. Giles’ indignation, therefore, might strike this individual as distasteful, insensitive, even offensive, because his indignation seems to come from the position of the political parvenu – someone whose own experience allows them to feel indignant disbelief that governments can restrict one’s freedoms. Giles’ indignation fails to take into account that although the moral injustice might be a nasty surprise for him, this injustice is the bedrock of everyday life for others.

To offer another example, say that Giles posts the next day his indignation about racism in the police force – “How dare the police carry out stop and search procedures based on racial profiling??”. While we might praise his anger and disgust about this state of affairs, his seeming disbelief about this happening seems inappropriate. We have the feeling that he should already be aware that this happens and that his surprise, and thus his indignation, is unwarranted. To only *now* be indignant about this suggests that Giles has been living in a bubble of privilege. We might find something naïve, even distasteful, about the way in which Giles’ world admits to this being a negatively valanced surprise. Giles’ indignation may rightly be disclosing a moral wrong but it simultaneously reveals that the world Giles inhabits seems notably ignorant of the long history and reality of racism and police violence.

Indeed, when people express indignant disbelief over on-going systemic moral offences, what might contribute to the feeling that such indignation is inappropriate is because the indignation may be propped up by, or grounded upon, epistemic injustice. For example, we might suspect that one could only occupy a world where the very occurrence of, say, structural racism begs belief, if that world is predicated on the testimony and experiences of certain individuals and groups not being allocated sufficient weight or credence (Fricker, 2007), such that their experiences might still seem surprising to other people. What might be offensive about indignation in relation to certain matters, is that it brings to the fore the continued ignorance of, or outright disinterest in, the struggles that marginalised people face in their everyday worlds.

Indignation, then, might strike us as the remit of the world-naïve or -ignorant rather than the worldly. People who occupy positions of relative privilege might seem much more likely to experience indignation and, in turn, their indignation often reveals that privilege.^15^ As indignation is experienced in relation to one’s own expectations and understanding of the world, even though one’s involvement might be accidental, one’s moral, social, and political situatedness is not. For to find moral injustices surprising or not depends upon one’s own understanding and sense of the world. Where that sense of the world is overly naïve, even ignorant, self-centred and lacking in awareness, indignation can be received as inappropriate and offensive.

### Holding onto indignation

If indignation is an emotion characterised by disbelief, there are occasions when holding onto indignation is very powerful. Refusing not to be surprised, even when our past experiences suggest otherwise, can be a forceful way of demanding more from the world. For a real-world example, take Greta Thunberg’s “How dare you!” speech that she gave at the 2019 United Nations Climate Action Summit:

This is all wrong. I shouldn’t be up here. I should be back in school on the other side of the ocean. Yet you all come to us young people for hope? How dare you! You have stolen my dreams and my childhood with your empty words. And yet I’m one of the lucky ones. People are suffering. People are dying. Entire ecosystems are collapsing. We are in the beginning of a mass extinction. And all you can talk about is money and fairytales of eternal economic growth. How dare you!^16^

This was by no means her first speech denouncing the political inaction of world leaders and governments in the face of the climate crisis. As such, we might suppose that Thunberg should not be surprised by this inaction, indeed, that she should expect precisely this. Yet, in refusing to let the reality dictate her expectations in favour of what she thinks should be a reasonable expectation (i.e., that world leaders and governments respond to this crisis with sincerity and urgency), Thunberg holds onto her indignation. Maintaining that the way world leaders and governments have acted *is* surprising and should continue to beg belief holds the world to better standards than what our experience might suggest. It rejects what is norm*al* for what should be the moral *norm*.

Yet, above, I suggested that to experience and express disbelief at the very real state of the world can be both naïve and offensive. What marks the Greta Thunberg case apart from my previous imagined example of Giles? I think the difference lies in the attitude of disbelief here. Importantly. Greta Thunberg’s indignation does not arise out of an ignorance of on-going injustice, it is not that she has been living in a bubbled world where she did not know this happened. Like in our earlier case of being indignant about a government’s on-going corruption, Thunberg’s disbelief is about the persistence of the morally unacceptable reaction of world leaders to climate crisis. Her disbelief is not born of naiveté, or a failure to accommodate other people’s experiences into one’s understanding of the world. It is born out of a refusal to allow our depressing reality to dictate what kind of world she thinks we should strive towards – a world where we do not sit in apathy as the world teeters on collapse with the world’s most vulnerable citizens closest to the edge. Thunberg’s disbelief, then, does not seem to be grounded in ignorance but in hope.

The pandemic might have revealed many occasions for indignation, such as governmental disinterest in protecting lives over economies, the exploitation and disregard of the vulnerable, the lack of gratitude and care of health workers, the impacts of poverty and discrimination on health. However, we should recognise the power and importance of remaining surprised about the occurrence of these injustices, of not letting their continued occurrence give way to resignation. Holding onto indignation, though, comes at a price. As Cherry (2022) highlights, maintaining any form of moral anger in the face of injustice is exhausting and is yet another form of emotional labour that is often shouldered by the marginalised and oppressed.

## Conclusion

Thinking through the lens of Covid-19, I have sought to highlight the distinctive character of indignation. In doing so, my first aim has been to contribute to broader discourse about how we think about anger and different forms of anger by refining our understanding of this emotion. My second aim, however, has been to reflect on how the character of indignation shapes how we perform and receive indignation. As I have argued, indignation is a complex emotion that can work to build our bonds with others, can be treated as narcissistic and smug, can even be offensive. However, I have also suggested that indignation, in being an emotion characterised in part by disbelief, can be a powerful political tool for refusing to accept the moral injustices of the world as they are and demanding more.

## Footnotes

1 For a recent discussion of emotions and evaluations, see Mitchell (2021).

2 For a discussion of specifically *political* anger, see Cherry (2022), Leboeuf (2018). For discussions about what makes an emotion a *political* emotion, see: Szanto & Slaby (2020), Osler and Szanto (2021).

3 Here I use the phrase “morally offensive” broadly and to also apply to political and social offences.

4 Though notice that it seems being aware that one’s indignation is hypocritical seems to undermine that indignation. In contrast, while I might be aware of being unfairly angry at the chip-taker for taking the last chip (given that I have eaten the majority of our chips already) this does not necessarily take the wind out of the sails of my anger.

5 For a discussion of whether surprise is, itself, an emotion, see: Depraz (2018).

6 For a distinction between surprise and shock, see Stockdale (2022).

7 I will discuss in Section 4 that in being an emotion of surprised disbelief, we might suppose that indignation is more commonly experienced by individuals with a certain degree of privilege.

8 A huge thank you to the anonymous reviewer who suggested this response.

9 In their discussion of blame, Shoemaker and Vargas (2021) suggest that a unifying feature of different types and expressions of blame is that they all serve the function of signalling the blamer’s commitment to various norms or values.

10 For a rich and important discussion of how people can inhabit different ‘worlds’, see Lugones 1987.

11 For discussions of how to conceive of online emotions, see Bortolan (2022) for a narrative account of online emotions and Candiotto (2022) for an extended account. For a more general discussion of how social interactions online regulate emotions and affect, see Krueger and Osler (2019).

12 For broader discussions of communal experiences online, see Froese et al. (2020), Osler (2020), Osler (2022b).

13 Here I use the word crisis in line with colloquial descriptions of the Covid-19 pandemic. For an analysis of how we should conceive of the term ‘crisis’ see Tietjen (2023) and a persuasive argument for moving away from crisis epistemology by Whyte (2021).

14 For a fascinating discussion of vice signalling, see Táíwò (2022).

15 That indignation is often associated with privilege also sheds some uncomfortable light on indignation’s role as an allyship emotion. Remember that while we can feel indignant in relation to a moral offence perpetrated against us, our own harm is not necessary. Gail might be indignant that a government is not financially supporting people during the pandemic, while being financially secure themselves. However, in being an emotion that can be had ‘on behalf of others’, indignation treads the complicated path on what it is to be an ally to others. For a wonderful discussion about allies and moral anger, see Cherry (2022).

16 Thunberg, Greta (23 September 2019). “If world leaders choose to fail us, my generation will never forgive them | Greta Thunberg”. The Guardian. ISSN 0261–3077. Archived from the original on 23 September 2019.
